# Potential for Ketotherapies as Amyloid-Regulating Treatment in Individuals at Risk for Alzheimer’s Disease

**DOI:** 10.3389/fnins.2022.899612

**Published:** 2022-06-16

**Authors:** Matthew K. Taylor, Debra K. Sullivan, Jessica E. Keller, Jeffrey M. Burns, Russell H. Swerdlow

**Affiliations:** ^1^Department of Dietetics and Nutrition, University of Kansas Medical Center, Kansas City, KS, United States; ^2^University of Kansas Alzheimer’s Disease Research Center, Fairway, KS, United States; ^3^Department of Neurology, University of Kansas Medical Center, Kansas City, KS, United States

**Keywords:** ketogenic diet, medium chain triglyceride (MCT), Alzheimer’s disease, amyloid, mitochondria, exogenous ketones, ketotherapy

## Abstract

Alzheimer’s disease (AD) is a progressive neurodegenerative condition characterized by clinical decline in memory and other cognitive functions. A classic AD neuropathological hallmark includes the accumulation of amyloid-β (Aβ) plaques, which may precede onset of clinical symptoms by over a decade. Efforts to prevent or treat AD frequently emphasize decreasing Aβ through various mechanisms, but such approaches have yet to establish compelling interventions. It is still not understood exactly why Aβ accumulates in AD, but it is hypothesized that Aβ and other downstream pathological events are a result of impaired bioenergetics, which can also manifest prior to cognitive decline. Evidence suggests that individuals with AD and at high risk for AD have functional brain ketone metabolism and ketotherapies (KTs), dietary approaches that produce ketone bodies for energy metabolism, may affect AD pathology by targeting impaired brain bioenergetics. Cognitively normal individuals with elevated brain Aβ, deemed “preclinical AD,” and older adults with peripheral metabolic impairments are ideal candidates to test whether KTs modulate AD biology as they have impaired mitochondrial function, perturbed brain glucose metabolism, and elevated risk for rapid Aβ accumulation and symptomatic AD. Here, we discuss the link between brain bioenergetics and Aβ, as well as the potential for KTs to influence AD risk and progression.

## Introduction

Alzheimer’s disease (AD) is a devastating neurodegenerative disease that is expected to affect more than 15 million Americans by the year 2050 (Colby and Ortman, 2014). There are several different proposed hypotheses to describe its underlying basis, the oldest and most studied of which is the “Amyloid Cascade Hypothesis” (Hardy and Higgins, 1992). This hypothesis suggests that the aggregation of amyloid-β (Aβ) peptides initiates the downstream symptoms and neurodegeneration associated with AD. Aβ accumulates for years prior to the onset of symptomatic AD and is currently considered a diagnostically obligatory component of the disease (Sperling and Johnson, 2013).

Impaired brain metabolism in the form of mitochondrial dysfunction and reduced glucose utilization has also long been recognized as an important AD hallmark. Like Aβ, impaired brain bioenergetics can occur long prior to AD clinical symptoms (Reiman et al., 2004; Mosconi et al., 2009) and mechanistic studies suggest impaired brain metabolism and Aβ influence each other in a complex, cyclical manner (Wilkins and Swerdlow, 2017). The reasons for Aβ accumulation in AD are currently unknown, and hypotheses designed to address this question exist. Examples include the propositions that impaired bioenergetics (Swerdlow and Khan, 2004) and conditions of chronic inflammation and oxidative stress (Decourt et al., 2022) over a lifetime may drive the disease and initiate its classic histopathologies. Such hypotheses do not assume Aβ triggers the disease. Rather, it has been hypothesized that altered Aβ homeostasis, manifesting as either increased production or decreased clearance, may be an important AD biomarker that actually reflects impaired mitochondrial bioenergetics (Wilkins et al., 2022).

An estimated 20–40% of cognitively unimpaired older adults have elevated cerebral Aβ, meeting the definition of “preclinical AD” (Dubois et al., 2016). These individuals exhibit brain hypometabolism, rapidly accumulate new Aβ, and are at greatly increased risk of developing symptomatic AD (Sperling et al., 2011; Lowe et al., 2014). Furthermore, it is thought that over half of the older adult population may have peripheral metabolic impairments (i.e., metabolic syndrome, insulin resistance, type 2 diabetes) (Denys et al., 2009), conditions increasingly linked to mitochondrial dysfunction and higher risk of AD-related neurodegeneration and cognitive decline (Morris et al., 2014b; Paul et al., 2021). For these reasons, both groups are ideal populations in which to test potential AD therapies that specifically target bioenergetic metabolism.

## Amyloid-β and Alzheimer’s Disease

### Amyloid as a Biomarker of Elevated Risk for Alzheimer’s Disease

Although a small proportion of AD diagnoses are familial, greater than 97% of all diagnoses are late onset, sporadic AD (Garcia-Morales et al., 2021). Recent efforts to define the continuum of AD pathology from asymptomatic preclinical AD to symptomatic, sporadic AD have elucidated characteristic biomarker changes throughout the spectrum of AD progression. Although brain bioenergetics can be impaired prior to symptomatic AD or prodromal mild cognitive impairment (MCI) (Reiman et al., 2004; Mosconi et al., 2009; Sperling and Johnson, 2013), brain accumulation of Aβ is often cited as the first hallmark biomarker in the progression from normal aging to the preclinical AD phase; yet, Aβ alone is not sufficient to manifest as symptomatic AD (Sperling and Johnson, 2013). Thus, the now widely accepted criterion for “preclinical AD” is defined as individuals that are asymptomatic for AD but positive for Aβ accumulation (Dubois et al., 2016). Aβ accumulation occurs most rapidly during this preclinical phase and slows after reaching relative saturation and onset of AD symptoms (Jack et al., 2013; Burgold et al., 2014). Whether Aβ is a downstream biomarker of AD pathology or an upstream initiator, elevated Aβ is an important indicator of increased risk for clinical AD.

The role for Aβ in normal physiology is not well understood. It is expressed in low concentration in multiple peripheral tissues and the brain throughout the lifespan. In the brain, Aβ monomers are thought to be expressed in response to neuroinflammation as part of the brain’s innate immune system (Van den Heuvel et al., 1999; Plummer et al., 2016) causing some to hypothesize elevated Aβ is a physiological response to excessive oxidative stress and neuroinflammation. Other putative roles for Aβ at low brain concentration include modulation of learning and memory (Morley et al., 2010), clotting of blood vessels along the blood brain barrier (Atwood et al., 2003), and antimicrobial and antiviral activity (Kumar et al., 2016; Eimer et al., 2018). Aβ peptides have a high affinity for binding to form dimers and oligomers; thus, overexpression of Aβ increases the likelihood of accumulating neurotoxic Aβ plaques.

### Synthesis of Amyloid-β

Aβ is a peptide, commonly of 40 (Aβ40) or 42 (Aβ42) amino acids in length in its primary forms (Takami et al., 2009; Olsson et al., 2014), derived from multiple cleavages of amyloid precursor protein (APP) by β- and γ-secretase (Nunan and Small, 2000). In AD, Aβ42 primarily forms the fibrillar Aβ plaques that accumulate in extracellular space of the brain with Aβ40 thought to contribute less to plaque formation (Jarrett et al., 1993). APP is expressed systemically across multiple cell types with high concentration found at the neuronal synapse where it acts as a regulator of synaptogenesis, synaptic repair, neuronal transport, and iron export (Turner et al., 2003; Priller et al., 2006; Duce et al., 2010). APP is synthesized intracellularly by mRNA-bound polysomes then transports to the endoplasmic reticulum, Golgi apparatus, and trans-Golgi network where it undergoes specific post-translational protein modifications within each organelle. Stepwise post-translational modifications regulate APP’s movement through its transport pathway, and after maturation, it is transported to the plasma membrane where it is cleaved by secretase enzymes (primarily α-, β-, and γ-secretases).

The majority of APP is processed in the “non-amyloidogenic” pathway where cleavage by α-secretase produces a secreted APPα (sAPPα) fragment that is released into extracellular space and an 83 amino acid C-terminal fragment (CTF83) that remains at the plasma membrane. CTF83 is cleaved by γ-secretase to form p3 (i.e., truncated Aβ peptide) that is released into extracellular space and an APP intracellular domain (AICD) peptide that enters the cytoplasm. The truncated Aβ peptide, p3, in AD has not been well studied and recent work suggests that p3 can aggregate in AD and that α-secretase cleavage of APP may not truly be “non-amyloidogenic” (Kuhn et al., 2020). Cleavage of APP by β-secretase, beta-site cleaving enzyme 1 (BACE1), at the Asp1 site initiates the “amyloidogenic” processing pathway, producing sAPPβ that is released extracellularly and CTF99 which remains in the plasma membrane. CTF99 is cleaved by γ-secretase to form AICD that enters the cytoplasm and Aβ that is either released to extracellular space or packaged into lipid rafts (Chen et al., 2017). It has also been suggested that organelles involved in the APP transport pathway express secretase enzymes and that co-residence of these enzymes with APP may result in some production of Aβ intracellularly (Wang et al., 2017).

### Brain Amyloid-β Clearance

Brain clearance of Aβ is important for regulation of Aβ concentration (Iwata et al., 2000) and occurs *via* multiple pathways including efflux and localized protein degradation. Approximately 50% of all brain Aβ clearance occurs *via* efflux with 25% directly transporting across the blood brain barrier and 25% entering cerebro-spinal fluid (CSF) for reabsorption into venous circulation (Roberts et al., 2014). In the process of transporting across the BBB, Aβ binds to protein chaperones such as Apolipoprotein E (ApoE) and transport is facilitated by lipoprotein receptor-related protein 1 (LRP1) (Cheng et al., 2020) and other supporting receptors (Bell et al., 2007; Hartz et al., 2010; Storck et al., 2018). Localized Aβ degradation also accounts for a significant amount of Aβ clearance, facilitated by microglia, and to a smaller degree, astrocytic phagocytosis (Fujita et al., 2020) and the enzymatic activity of neprilysin, insulin degrading enzyme, and angiotensin converting enzyme (Cheng et al., 2020). APP and Aβ can also localize within the mitochondrial matrix and expression of Aβ degrading enzymes within the mitochondrial matrix facilitate inner-mitochondrial Aβ clearance (Falkevall et al., 2006; Pinho et al., 2014).

### Alterations in Amyloid-β Synthesis and Clearance in Alzheimer’s Disease

Regulation of Aβ synthesis and clearance are perturbed in AD, resulting in increased accumulation of Aβ in cerebral plaques.

In familial AD, Aβ accumulation is attributed to altered APP processing due to one or more mutations in the genes that encode for APP and presenilin 1 and 2 (PS1 and PS2) (Silva et al., 2019). Genetic mutation of the APP gene increases APP’s susceptibility to amyloidogenic BACE1 cleavage (Zhang et al., 2017). PS1 and PS2 comprise one of the four subunits of γ-secretase and mutation of PS-encoding genes often increases γ-secretase activity and Aβ42 production (Bentahir et al., 2006).

Although several genes have been linked as risk factors for sporadic AD, allelic expression of Apolipoprotein E ε4 (APOE ε4) is thought to be the most impactful genetic risk factor. Approximately 9–23% of humans express at least one APOE ε4 allele (Jia et al., 2020), a prevalence that varies by race and ethnicity, which increases AD risk with even higher risk in APOE ε4 homozygotes (Serrano-Pozo et al., 2011). Human APOE ε4 carriers consistently demonstrate higher Aβ burden and more rapid accumulation than non-carriers (Tiraboschi et al., 2004; Drzezga et al., 2009; Caselli et al., 2010; Rowe et al., 2010; Baek et al., 2020), likely due to various effects including increased transcription of APP, potentially altered γ-secretase activity, interrupted Aβ degradation, and poor transport of Aβ across the blood brain barrier (Huang et al., 2017, 2019; Wong et al., 2020). We discuss the link between impaired brain bioenergetics and Aβ later, but individuals with ApoE ε4 exhibit brain hypometabolism as early as young adulthood (Reiman et al., 2001, 2004; Mosconi et al., 2008a; Murray et al., 2014), which implicates impaired bioenergetics as a factor that mediates the influence of ApoE on Aβ. Though carrying APOE ε4 is the strongest genetic risk factor for sporadic AD, less than 50% of individuals that are diagnosed with AD are APOE ε4 carriers (Crean et al., 2011), indicating that other factors are involved in AD etiology.

Aberrant APP post-translational modification and trafficking are also observed in patients with sporadic AD (Lee et al., 2003; Placido et al., 2014; Joshi and Wang, 2015) which alter the fate of APP and has been thoroughly reviewed (Wang et al., 2017). The factors involved in dysregulated APP processing and upregulation of Aβ generation in sporadic AD are not well understood, but they may include impairments in mitochondrial function and bioenergetics, polymorphisms or mutations in other responsible genes, environmental exposures, and lifestyle behaviors. A likely scenario is that an interaction among these suggested factors influences APP processing, Aβ production, and AD risk.

### Bioenergetics, Amyloid Precursor Protein Processing, and Amyloid-β

Mitochondria are the primary sites for cellular energy metabolism. APP, its processing, and mitochondria exhibit a complex, dynamic relationship where dysregulation of either APP or mitochondria exert detrimental influential effects upon the other in a vicious cycle. Here we briefly review this relationship as this topic has been previously reviewed in detail (Wilkins and Swerdlow, 2017).

Mitochondrial bioenergetics are influenced by dysregulated mitochondrial import of APP and Aβ. APP localized to the mitochondria is thought to inhibit mitochondrial protein import (Gottschalk et al., 2014) and alter respiratory chain function (Devi et al., 2006). However, alterations to APP that reduce the amount that localizes to the mitochondria have also been shown to impair mitochondrial function (Wang et al., 2016), suggesting that adequate, but not excessive, APP localization is important for maintaining mitochondrial integrity. On the other hand, increased APP accumulation has been observed in the brain mitochondria of humans with confirmed AD (Devi et al., 2006) and overexpression of APP is linked with increased Aβ within the mitochondria (Rhein et al., 2009). Mitochondrial expression of Aβ is consistently linked to decreased cellular respiration, cytochrome oxidase (COX) activity, and ATP production along with increased production of reactive oxygen species (ROS) (Rhein et al., 2009; Pinho et al., 2014). Mitochondria are also reported to express the γ-secretase enzyme (Area-Gomez et al., 2009; Pavlov et al., 2011) and mitochondrial localization of the APP-derived CTF99 protein fragment (which is cleaved by γ-secretase to form Aβ) is upregulated in AD (Pera et al., 2017). Increased CTF99 may directly exert detrimental effects upon the mitochondrial respiratory chain in addition to detrimental effects of Aβ in AD. Aβ also diminishes brain glucose uptake by activating membrane-bound NADPH oxidase (NOX), an enzyme responsible for ROS production, to overproduce ROS that is released into cytosol and can damage mitochondria (Malkov et al., 2021).

Conversely, glucose metabolism and mitochondrial bioenergetics influence APP processing and its fate. Many *in vitro* studies suggest that inhibiting glycolysis or mitochondrial respiration downregulates non-amyloidogenic APP processing pathways (Gasparini et al., 1997, 1999), upregulates Aβ synthesis (Fu et al., 2015), leads to APP trapping in the ER and Golgi (Gabuzda et al., 1994; Domingues et al., 2007), and further exacerbates bioenergetic perturbations of glycolysis and mitochondrial respiration (Swerdlow et al., 2013; Korenic et al., 2014). In cytoplasmic hybrid (cybrid) cells, cell models allowing for the study of various effects of mitochondrial DNA (mtDNA) (Wilkins et al., 2014), transferring mtDNA from patients with sporadic AD reduced ATP production and COX activity (Khan et al., 2000; Cardoso et al., 2004) and increased ROS (Cardoso et al., 2004) and Aβ (Khan et al., 2000) production compared to mtDNA from age-matched controls. Mitochondria with low bioenergetic potential have increased APP translocation and express more Aβ, suggesting that APP targets these impaired mitochondria and that decreased fluid Aβ42/Aβ40 ratio may actually be a biomarker for impaired mitochondrial bioenergetics (Wilkins et al., 2022). Increased Aβ production within the mitochondria also results in higher inner mitochondrial AICD, which is neurotoxic in a manner independent of Aβ (Sandberg et al., 2022). *In vivo* studies in mouse models suggest that APP processing can be altered *via* bioenergetic manipulation. In transgenic mice with APP gene mutations, increasing antioxidant activity reduced Aβ production (Mao et al., 2012) and cortical plaques (Dumont et al., 2009; Mao et al., 2012) while decreasing antioxidant activity increased Aβ production and plaque formation (Li et al., 2004). Higher mitochondrial production of ATP is also related to fewer and smaller Aβ plaques (Scheffler et al., 2012) and inhibition of ATP production results in upregulated ROS and Aβ (Leuner et al., 2012). This suggests that bioenergetics and ROS are involved in APP processing and Aβ generation.

Clearance of Aβ is an energy-dependent process, which not only implies poor mitochondrial bioenergetics are related to increased Aβ production, but also reduced Aβ clearance. Increased mitochondrial ATP production is linked to increased Aβ plaque clearance (Scheffler et al., 2012) and impaired mitochondrial function due to accumulation of mtDNA mutations decreases Aβ clearance (Kukreja et al., 2014).

## Ketotherapies

We have previously reviewed KT approaches in detail (Taylor et al., 2019b). Briefly, KTs are dietary approaches that promote ketogenesis, the synthesis of ketone bodies, with the goal of substituting or augmenting brain glucose metabolism. For the sake of this review, we will discuss the ketogenic diet (KD), medium-chain triglycerides (MCT), and exogenous ketone supplements.

### Ketogenic Diet

The ketogenic diet (KD) is an eating pattern characterized by high fat, very low carbohydrate, and adequate protein intake. Americans typically consume roughly half of their energy intake as carbohydrate (Shan et al., 2019) which converts to glucose as the primary substrate for energy metabolism. By reducing carbohydrate intake to < 10% of total energy and increasing fat intake as the primary (65–70%) dietary macronutrient, glucose metabolism is insufficient to support global energy status and the body shifts to ketogenesis for energy production.

The KD induces a major metabolic shift where reduction of dietary carbohydrate intake physiologically decreases circulating insulin and increases circulating glucagon (Apfelbaum et al., 1972) to promote the breakdown of stored glucose (glycogenolysis) and the production of *de novo* glucose (gluconeogenesis) to maintain glucose homeostasis (Koppel and Swerdlow, 2018; Zhang et al., 2018). Once endogenous glucose stores are depleted, as in the case of the KD, increased beta-oxidation of fatty acids in the hepatic mitochondria produces an abundance of 2-carbon acetyl Co-A. Acetyl Co-A production exceeds the metabolic capacity of the tricarboxylic acid (TCA) cycle and carbon moieties are shuttled into the ketogenic pathway where, through several reactions, they are converted to the primary ketone bodies acetoacetate (AcAc) and beta-hydroxybutyrate (BHB). BHB is the most stable of the ketone bodies and is found in the most abundance in circulation compared to AcAc and its decarboxylated byproduct, acetone (Ac) (Dabek et al., 2020). Ketone bodies in the blood are typically < 0.1 mmol/L in the presence of dietary carbohydrate (Gershuni et al., 2018). Nutritional ketosis induced by a KD results in levels generally between 0.5 and 1.5 mmol/L, but can safely reach levels higher than 3 mmol/L (Miller et al., 2018).

The recent emergence of the well-formulated KD (WFKD), which we have shown can be nutritionally dense (Taylor et al., 2019a; Rippee et al., 2020), seeks to address a common criticism that KDs lack diet quality and variety. These arguments likely stem from formative KD work in epilepsy (Martin-McGill et al., 2018), where strict classical 4:1 KD ratios, a ratio of 4 g of fat consumed for every 1 g of carbohydrate and protein combined (i.e., 90% energy from fat and ≤ 2% energy from carbohydrate), leave little room for nutrient-dense foods in order to meet macronutrient goals for epileptic seizure management. The WFKD generally reduces the KD ratio to ∼1:1 KD ratio, a ratio of 1 g of fat consumed for every 1 g of carbohydrate and protein combined (i.e., 70% energy from fat and ≤ 10% energy from carbohydrate); emphasizes diet quality by including intake of whole, real foods such as non-starchy vegetables, healthy fats (e.g., nuts, seeds, avocado), and healthy proteins (e.g., omega-3 containing fish); and is capable of producing robust ketosis.

### Medium-Chain Triglycerides

Medium-chain triglycerides (MCTs) are comprised of glycerol backbones with aliphatic fatty acid tails that are 6–12 carbons in length and are more readily absorbed than long-chain triglyceride (LCT) (Liu and Wang, 2013; Barzegar et al., 2021). Unlike LCTs that require passage through the lymphatic system for delivery to the liver, MCTs are absorbed directly into the portal blood, enter the liver to be rapidly metabolized to ketone bodies, and are transported to extrahepatic tissue for mitochondrial uptake. MCT-containing products can have various fatty acid compositions, though most typically provide a majority caprylic acid (C8) and capric acid (C10) (Augustin et al., 2018). MCTs alone produce an acute robust ketosis (Barzegar et al., 2021), therefore individuals who supplement with MCTs can enter ketosis without following a KD (Liu and Wang, 2013). MCT-supplemented KDs allow for a more liberal KD that can enhance palatability, variety, and nutrient intake (Liu and Wang, 2013).

MCTs are naturally occurring in coconut oil, coconut products, palm kernel oil, and whole dairy products and marketed products can be derived from any one of these foods or synthetically made. Since MCTs are directly absorbed in the gut rather than undergoing emulsification in bile salts, large doses of MCT can cause GI distress. Emulsification of MCTs is suggested to optimize absorption, GI tolerance, and ketone production (Courchesne-Loyer et al., 2017b) which has led to a recent rise in availability of emulsified MCT products.

### Exogenous Ketone Supplements

Exogenous sources of ketones are another way to induce systemic ketosis without dietary macronutrient manipulation (Brunengraber, 1997). Though exogenous ketones exist in the form of ketone salts, ketone esters (KE), primarily ketone monoesters, are demonstrated to be most tolerable and ketogenic in humans (Shivva et al., 2016; Stubbs et al., 2017). KEs are constituted of R-1,3-butanediol and BHB that are cleaved by gut esterases and enter hepatocytes *via* the portal vein (Clarke et al., 2012). R-1,3-Butanediol is converted to BHB in the hepatocyte by aldehyde dehydrogenase (Desrochers et al., 1995), resulting in hepatic mitochondrial import of two BHB molecules.

## Ketotherapies and Amyloid-β

Preliminary studies suggest that KTs may benefit cognition in patients with AD and MCI (Henderson et al., 2009; Krikorian et al., 2012; Taylor et al., 2018; Fortier et al., 2019). Many narrative reviews of this topic have been published, and more recently, attention has turned toward use of KTs as potential approaches in the prevention of AD (Davis et al., 2021). Here, we highlight the current evidence for KTs as modulators of Aβ and AD biology.

### Evidence in Animal and Human Studies

There is evidence that KTs may beneficially affect Aβ in both transgenic AD-model and non-transgenic mice as well as in humans at risk for AD. Compared to a standard chow diet, APP/V717I transgenic mice fed a 43-day KD had a 25% reduction in brain Aβ levels (Van der Auwera et al., 2005). Similarly, 5xFAD mice fed a 4-month KD had reduced hippocampal Aβ deposition compared to the standard chow diet (Xu et al., 2021). Contrary to these findings, studies of a 16-week (Brownlow et al., 2013) and 1-month (Beckett et al., 2013) KD in APP/PS1 transgenic mice demonstrated improvements in motor function but not Aβ deposition; however, the APP/PS1 mice on the 1-month KD had decreases in brain and skeletal muscle CTF99 (Beckett et al., 2013), the precursor for Aβ. 3xTg-AD mice fed an 8-month 43.5% carbohydrate diet supplemented with 21.5 g of ketone ester reduced Aβ and tau and improved memory and anxiousness compared to a 64.9% carbohydrate diet without ketone ester (Kashiwaya et al., 2013). APP mutant mice that received daily 600 mg ketone body injections for 2 months had fewer Aβ plaques and decreased soluble and insoluble Aβ42 expression relative to mice that received normal saline injection (Yin et al., 2016). In non-transgenic mice injected with soluble Aβ, 8-weeks of intermittent fasting induced mild ketosis and was protective of hippocampal Aβ deposition, whereas the experimental KD did not protect against Aβ deposition (Park et al., 2020). Finally, a 6-week KD in 20 humans with either MCI or subjective memory complaints increased Aβ42 levels in cerebral spinal fluid (Neth et al., 2020), indicating a likelihood of better Aβ clearance and less contribution to cerebral plaque formation. Together, these studies suggest that the KTs may improve APP processing and Aβ clearance.

### Putative Mechanisms of Ketotherapies and Amyloid-β

**FIGURE 1:**
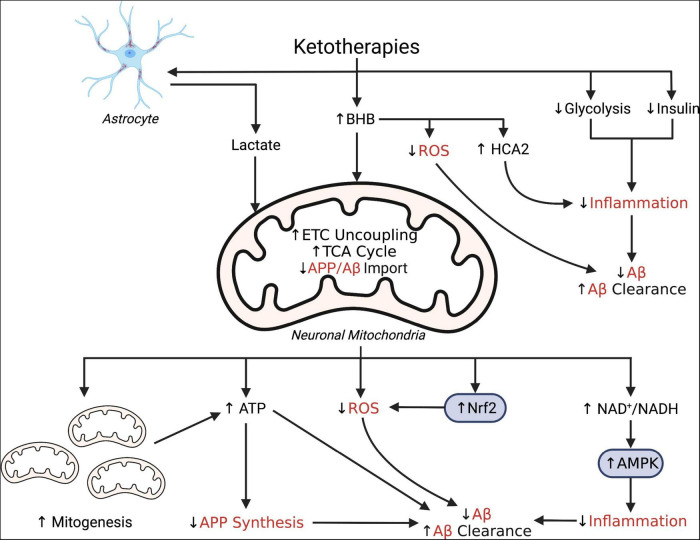
Simplified schematic illustrating putative pleiotropic effects of ketotherapies as modulators of amyloid-β. Ketotherapies (KTs) potentially modulate amyloid-β (Aβ) through various direct and indirect mechanisms targeting poor mitochondrial bioenergetics, increased ROS, and increased inflammation. KTs, especially the ketogenic diet (KD), reduce systemic insulin and potentially improve peripheral metabolic status which may improve systemic inflammation and reduce Aβ. The ketone body, β-hydroxybutyrate (BHB), serves as an energy substrate for mitochondrial metabolism, upregulates the astrocyte-neuron lactate shuttle, activates hydrocarboxylic acid receptor 2 (HCA2) to regulate inflammation, and may directly scavenge reactive oxygen species (ROS). Through bioenergetic effects in the mitochondria, KTs stimulate genesis of new mitochondria, increase uncoupling of the electron transport chain (ETC) to increase ATP production, generate less ROS than glucose metabolism, and reduce mitochondrial import of amyloid precursor protein (APP) and Aβ. KTs also activate nuclear factor-E2 related factor 2 (Nrf2) to upregulate synthesis of ROS-scavenging antioxidants and AMP-activated protein kinase (AMPK) to regulate transcription of pro-inflammatory cytokines. This figure created with BioRender (https://biorender.com).

The reason that Aβ is dysregulated in the pathological process leading up to symptomatic AD is still not well understood. Several AD etiological hypotheses suggest that impaired bioenergetics could explain this phenomenon. For instance, the mitochondrial cascade hypothesis for Alzheimer’s disease suggests that AD pathology, including dysregulation of Aβ, is due to impaired mitochondrial bioenergetics (Swerdlow and Khan, 2004). AD is also increasingly linked to several chronic conditions of inflammation and oxidative stress that that are known to influence brain glucose metabolism, mitochondrial function, and Aβ, which indicates that AD may be a condition of multiple pathologies (Decourt et al., 2022) and not just of the brain alone (Morris et al., 2014a). Altered bioenergetics in the form of impaired brain and systemic glucose energy metabolism, whether induced by oxidative stress, impaired mitochondria, or accumulation of Aβ, is consistently observed in patients with symptomatic AD and up to several decades prior to onset of symptoms (Ferris et al., 1980; de Leon et al., 1983, 2001; Foster et al., 1983; Friedland et al., 1983; Ishii et al., 1997; Reiman et al., 2004; Mosconi et al., 2008a,b, 2009; Rosenbloom et al., 2011; Castellano et al., 2015). Here, we have highlighted the link between changes in bioenergetics and Aβ and suggest that, through pleiotropic effects, KTs may hold potential as amyloid-regulating therapies. These potential mechanisms are also illustrated in Figure 1.

#### Improved Brain Metabolism

KTs may bolster overall brain metabolism by providing an alternative fuel substrate when glucose metabolism is impaired, potentially exerting beneficial effects on APP processing. In an attempt to explain decreased glucose uptake observed in AD, it is commonly hypothesized that neuronal death and decreased synaptic activity result in decreased glucose demand (Blass and Zemcov, 1984), even though this phenomenon is observed prior to substantial neurodegeneration in the timeline of AD biomarker progression. On the other hand, patients with AD and MCI are able to use ketone bodies for brain energy metabolism (Lying-Tunell et al., 1981; Swerdlow et al., 1989; Ogawa et al., 1996; Castellano et al., 2015) where increased brain ketone uptake is proportional to ketone substrate availability (Courchesne-Loyer et al., 2017a; Croteau et al., 2018; Fortier et al., 2019). Given this evidence, it is also plausible that brain ketone metabolism is viable in the AD preclinical phase. It is possible that ketone bodies could be used as an AD treatment or preventive strategy by partially rescuing impaired brain glucose metabolism (Blass and Zemcov, 1984) to regulate Aβ production if it is indeed induced by deficits in brain bioenergetics.

There are several mechanisms by which KTs may beneficially influence bioenergetics through mitochondrial metabolism of ketone bodies. The majority of AcAc and BHB are produced in the liver mitochondria, transport across the BBB, and enter mitochondria of the brain CNS where they undergo ketolysis for ATP production in the TCA cycle and electron transport chain (Taylor et al., 2019b). In the brain, evidence suggests that ketone bodies uncouple mitochondrial respiration (Sullivan et al., 2004; Liu et al., 2006; Kashiwaya et al., 2010), enhance multiple complexes of the mitochondrial respiratory chain (Ho et al., 2006; Chu et al., 2009; Wei et al., 2009; Kwok et al., 2010; Ramsden et al., 2012), and increase ATP production (Ho J. W. et al., 2012; Ho P. W. et al., 2012). The KD has also been shown to modify expression of genes associated with neurodegenerative disease, including counteracting impairments in oxidative phosphorylation (Koppel et al., 2021) and improving expression of metabolism-related genes in the hippocampus (Ling et al., 2019), a region susceptible to Aβ accumulation in AD pathology (Oh et al., 2016). A study of the KD supplemented with MCTs reportedly stimulated the astrocyte-neuron lactate shuttle (Augustin et al., 2018) allowing the neuron to import more lactate for conversion to pyruvate and, subsequently, ATP (Magistretti and Allaman, 2018). It is also suggested that KTs may increase the number of mitochondria through activating pathways that regulate mitogenesis (Hughes et al., 2014; Hasan-Olive et al., 2019). Furthermore, ketosis in individuals with MCI (Fortier et al., 2019) and AD (Croteau et al., 2018) did not further reduce already diminished brain glucose uptake, thus it has been hypothesized that mitochondrial ketone metabolism spares available glucose substrates to be used for brain functions that uniquely require cytosolic metabolism of glucose (Zilberter and Zilberter, 2020).

#### Protection From Reactive Oxygen Species and Neuroinflammation

KTs may also regulate Aβ by mitigating the damaging effects of ROS. ROS are common byproducts of normal metabolic functions that play an important role in cellular physiology at homeostatic concentration but cause oxidative stress in excessive concentration (Miller et al., 2018). Excessive ROS is observed early in AD pathology (Wang et al., 2014) and markers of oxidative stress correlate well with Aβ levels in the brain (Butterfield et al., 1999; Butterfield and Lauderback, 2002; Sultana et al., 2006). In the case of impaired metabolism, dysfunctional mitochondria (Bonda et al., 2014; Wang et al., 2014) and NOX (Tarafdar and Pula, 2018) are major sources of excessive ROS production which can exacerbate metabolic impairments and further elevate ROS concentration (Wilkins and Swerdlow, 2017). KTs purportedly activate nuclear factor-E2 related factor 2 (Nrf2) (Milder et al., 2010; Lu et al., 2018), increasing synthesis of the antioxidants manganese superoxide dismutase (mnSOD) (Shimazu et al., 2013; Newman and Verdin, 2014) and glutathione (Jarrett et al., 2008) that scavenge ROS and protect against oxidative stress and mitochondrial damage. In healthy rats, the KD’s activation of hippocampal Nrf2 was apparently an adaptive response to reduce initial KD-related, acute increases in H_2_O_2_, a species of ROS (Milder et al., 2010). Whether this or other mechanisms may be associated with KT-induced Nrf2 upregulation in conditions with already high oxidative stress, such as preclinical AD, is unclear; yet, KTs are consistently linked to Nrf2 activation and decreased ROS in nervous tissue in such conditions (Cooper et al., 2018; Seira et al., 2021). In preclinical AD, hypometabolism induced by elevated Aβ may activate NOX (Shelat et al., 2008; Tarafdar and Pula, 2018; Abramov et al., 2020; Malkov et al., 2021), which has been hypothesized to be the prominent producer of ROS in pathologic conditions (Munro and Pamenter, 2019). The potential glucose-sparing action of KTs may divert glucose-6-phosphate into the pentose phosphate pathway to produce NADPH and maintain cytosolic antioxidant status (Zilberter and Zilberter, 2020). It is also suggested that mitochondrial metabolism of ketone bodies produces less ROS than glucose (Prins, 2008; Achanta and Rae, 2017), possibly related to upregulated uncoupling in mitochondrial respiration (Hansford et al., 1997; Votyakova and Reynolds, 2001; Echtay, 2007). BHB also functions as an antioxidant to directly scavenge ROS (Haces et al., 2008). KTs may reduce entry of small ROS particles into the inner mitochondrial membrane by inhibiting the mitochondrial permeability transition (mPT) pore (Emerit et al., 2004; Maalouf et al., 2009) and may also prevent mitochondrial import of Aβ and exacerbation of mitochondrial dysregulation and ROS production (Yin et al., 2016). High levels of ROS oxidize proteins like LRP1 to reduce its Aβ clearance activity (Owen et al., 2010), therefore, oxidative stress reduction induced by KTs may preserve their function.

KTs have also been shown to potentiate anti-inflammatory pathways which may protect against Aβ pathology by modulating neuroinflammation. Inflammation sensed by microglia and astrocytes in the CNS stimulates neuronal expression of inflammatory cytokines and Aβ (Frost et al., 2019; Liu et al., 2020). There are several ways in which KTs may modulate microglial activation and inflammatory response. Animal studies suggest that KTs increase the NAD^+^/NADH ratio in the brain (Grabacka et al., 2016; Elamin et al., 2018; Xin et al., 2018), which has been demonstrated to regulate transcription of pro-inflammatory cytokines (Shen et al., 2017). BHB may modulate neuroinflammatory response by activating microglial hydrocarboxylic acid receptor 2 (HCA2) (Zandi-Nejad et al., 2013; Rahman et al., 2014; Selfridge et al., 2015) and to downregulate the NLRP3 inflammasome and reduce pro-inflammatory cytokines IL-1 and IL-18 (Youm et al., 2015; Yamanashi et al., 2017; Trotta et al., 2019). It is suggested that the KD activates AMP-activated protein kinase (AMPK) (Harun-Or-Rashid and Inman, 2018) and, in turn, reduces NF-kB activation and transcription of pro-inflammatory cytokines (Nunes et al., 2015). Of note, elevations of Aβ have also been shown to stimulate pro-inflammatory cytokine expression by microglia, which may be more prominent in early stages of pathology while Aβ oligomers are still soluble rather than deposited into fibrillar plaques (White et al., 2005; Sondag et al., 2009). The KD has also been shown to decrease inflammatory microgliosis in several models including the 5xFAD mouse model (Xu et al., 2021).

#### Improved Glucoregulation

Elevated glucose, insulin resistance, and type 2 diabetes induce chronic inflammation and oxidative stress and are increasingly linked to elevated risk for AD (Khan and Hegde, 2020). Human studies suggest that consuming a high glycemic diet (Taylor et al., 2017, 2021) and worsening glucoregulation (Morris et al., 2016; Gomez et al., 2018; Honea et al., 2022) are related to increased Aβ burden in cognitively normal older adults, even in those without diabetes. Individuals with diabetes that have higher peripheral fasting glucose levels also have higher brain glucose levels, which is suggested to alter brain Aβ processing and clearance (Heikkila et al., 2009; Madhusudhanan et al., 2020). For instance, exposure of hyperglycemia in multiple cell types increases ROS levels (Nishikawa and Araki, 2007; Shenouda et al., 2011; Lee et al., 2016), reduces mitochondrial function (Nishikawa and Araki, 2007; Shenouda et al., 2011; Huang et al., 2022), reduces oligomeric Aβ clearance (Huang et al., 2022), and increases BACE1 expression and Aβ production (Lee et al., 2016). Interestingly, decreases in BACE1 have been shown to improve cellular glucose uptake (Hamilton et al., 2014), which is impaired in both diabetes and AD. Patients with AD exhibit brain insulin resistance (Messier and Teutenberg, 2005), similar to diabetes, which is thought to blunt neuronal glucose uptake, starve neuronal cells, impair mitochondrial bioenergetics, and increase ROS and neuronal Aβ production (Berlanga-Acosta et al., 2020). Insulin resistance and impaired glucose metabolism in the brain may alter glycosylation of APP, an important post-translational modification in the APP processing pathway, and favor Aβ formation (Liu et al., 2009; Chun et al., 2015). KTs, particularly the KD, reduce not only peripheral glucose and insulin resistance in humans (Yuan et al., 2020), but also neuronal insulin resistance in mice (Koppel et al., 2021), which could be useful to beneficially modulate Aβ production and clearance in individuals with metabolic risk factors for AD.

## Conclusion

A culmination of work across various cellular and living models suggests that KTs may be valuable for improving brain bioenergetics and modulating Aβ. Much of this work has been conducted using *in vitro* models of mitochondrial distress and AD transgenic mouse models, though some positive preliminary data in humans at risk for AD do exist. Humans with metabolic risk factors or preclinical AD due to elevated Aβ also exhibit impaired brain bioenergetics and susceptibility for Aβ accrual; and thus, are prime candidates to study prevention strategies that may physiologically change AD pathology. Given the current evidence highlighted in this narrative, KTs warrant investigation into their value for regulating Aβ in at risk individuals. Successful rescue of brain bioenergetics and reduction of Aβ in these individuals could potentially prevent downstream manifestation of other AD hallmarks such as tau, neurodegeneration, and cognitive impairment.

## Author Contributions

MT conceived, developed the manuscript, and took responsibility for the final content of the manuscript. DS, JK, JB, and RS provided critical revision for important intellectual content. All authors read and approved the final manuscript.

## Conflict of Interest

The authors declare that the research was conducted in the absence of any commercial or financial relationships that could be construed as a potential conflict of interest.

## Publisher’s Note

All claims expressed in this article are solely those of the authors and do not necessarily represent those of their affiliated organizations, or those of the publisher, the editors and the reviewers. Any product that may be evaluated in this article, or claim that may be made by its manufacturer, is not guaranteed or endorsed by the publisher.
